# Interconnections between m^6^A RNA modification, RNA structure, and protein–RNA complex assembly

**DOI:** 10.26508/lsa.202302240

**Published:** 2023-11-07

**Authors:** Simone Höfler, Olivier Duss

**Affiliations:** Structural and Computational Biology Unit, EMBL Heidelberg , Heidelberg, Germany

## Abstract

Protein–RNA complexes exist in many forms within the cell, from stable machines such as the ribosome to transient assemblies like the spliceosome. All protein–RNA assemblies rely on spatially and temporally coordinated interactions between specific proteins and RNAs to achieve a functional form. RNA folding and structure are often critical for successful protein binding and protein–RNA complex formation. RNA modifications change the chemical nature of a given RNA and often alter its folding kinetics. Both these alterations can affect how and if proteins or other RNAs can interact with the modified RNA and assemble into complexes. N^6^-methyladenosine (m^6^A) is the most common base modification on mRNAs and regulatory noncoding RNAs and has been shown to impact RNA structure and directly modulate protein–RNA interactions. In this review, focusing on the mechanisms and available quantitative information, we discuss first how the METTL3/14 m^6^A writer complex is specifically targeted to RNA assisted by protein–RNA and other interactions to enable site-specific and co-transcriptional RNA modification and, once introduced, how the m^6^A modification affects RNA folding and protein–RNA interactions.

## Abstract (precis)

This review summarizes current knowledge and future directions on the mechanisms that govern the interplay between m^6^A RNA modification, RNA folding, and protein–RNA interactions, focusing on the mechanisms and available quantitative information.

## Introduction

Protein–RNA complex assemblies are at the core of many essential cellular machineries, such as the spliceosome and the ribosome. In recent years, protein–RNA complexes have gained importance in more cellular processes, and studying them has become increasingly relevant to understanding the regulation of many cellular processes.

Similarly, since the discovery of the first modified nucleotide (pseudouridine) in yeast tRNA (Davis & Allen, 1957), the list of RNA modifications has not only grown in number and diversity but also their impact and role in regulating cellular processes have increasingly been appreciated and have become an integral factor in how RNAs and RNPs function and, for the latter, also how they assemble (Gilbert & Nachtergaele, 2023).

New and advancing technologies enable the discovery and characterization of currently around 170 chemically distinct RNA modifications on highly abundant RNAs such as ribosomal RNA (rRNA) and on rare and short-lived species including mRNAs, a subset of long noncoding RNAs (lncRNAs) and miRNAs (Dunin-Horkawicz et al, 2006; Czerwoniec et al, 2009; Machnicka et al, 2013; Boccaletto et al, 2018, 2022). These modifications and the enzymatic processes involved have context-dependent consequences on the affected RNA species, including effects on structure, stability, and ability to bind protein interaction partners (Watkins & Bohnsack, 2012; Motorin & Helm, 2022).

N^6^-methyladenosine (m^6^A) is among the best-studied internal RNA modifications found mainly on RNA polymerase II (Pol II) transcripts, including mRNAs, lncRNA, and primary microRNAs (pri-miRNA) (Alarcón et al, 2015b; Ke et al, 2017; Knuckles et al, 2017; Slobodin et al, 2017; Hong et al, 2022). Within mRNAs, m^6^A modifications are detected predominately in long introns and exons, terminal exons, and around stop codons (Dominissini et al, 2012; Meyer et al, 2012; Ke et al, 2015). m^6^A is prominently involved in almost all stages of gene expression, including transcriptional regulation, splicing, mRNA export, mRNA degradation, and translation (Meyer et al, 2015; Xiao et al, 2016; Barbieri et al, 2017; Roundtree et al, 2017; Slobodin et al, 2017; Zaccara et al, 2019).

Installation of the m^6^A modifications happens mainly co-transcriptionally by functionally distinct methyltransferases, also termed m^6^A writers, which each display different substrate specificities (Ke et al, 2017; Knuckles et al, 2017; Slobodin et al, 2017; Warda et al, 2017; Louloupi et al, 2018; Akichika et al, 2019; Ma et al, 2019; Sendinc et al, 2019; van Tran et al, 2019; Lee et al, 2021; Xu et al, 2022). The heterodimeric complex formed by methyltransferase-like protein 3 (METTL3) and METTL14 has a relatively broad range of substrates. It is responsible for most of the m^6^A marks on Pol II transcripts (Balacco & Soller, 2019). Nevertheless, METTL3/METTL14 requires the coordinated assembly of a complex multi-component RNP to ensure recruitment to target sites and correct modification of individual RNAs (Ping et al, 2014; Schöller et al, 2018; Wen et al, 2018; Yue et al, 2018; Balacco & Soller, 2019; Bawankar et al, 2021; Sepich-Poore et al, 2022; Su et al, 2022).

Other m^6^A-methyltransferases such as METTL16, METTL5/TRMT112, ZCCHC4, and PCIF/CAPAM have narrower substrate requirements and methylate only a very specific set of target RNAs and sequence contexts, but their recruitment to target sites relies, in most cases, on often short-lived but highly coordinated protein–RNA complex assemblies (Table 1) (Warda et al, 2017; Akichika et al, 2019; Ma et al, 2019; Sendinc et al, 2019; van Tran et al, 2019; Sendinc & Shi, 2023). These specialized m^6^A methyltransferases have been reviewed elsewhere (Huang et al, 2021; Yu et al, 2021b; Satterwhite & Mansfield, 2022) and will not be discussed here.

**Table 1.** Substrate specificities and kinetic parameters in m^6^A RNA modification for writers, readers, and erasers.

Writers	Substrate	K_m_ (Substrate)	K_m_ (SAM)	K_D_^(1)^	K_D_^(2)^	k_cat_	References
METTL3/METTL14	DRACH (D = A,G,U; R = A,G; H = A,C,U)	22 ± 2 nM	102 ± 15 nM	n/a	n/a	18 ± 2 h^−1^	Liu et al (2014) and Li et al (2016)
WTAP/VIRMA	human ACTB mRNA fragment	n/a	n/a	826.3 ± 336.0 nM	n/a	n/a	Su et al (2022)
WTAP/VIRMA/HAKAI	human ACTB mRNA fragment	n/a	n/a	562.7 ± 76.6 nM	n/a	n/a	Su et al (2022)
WTAP/VIRMA/ZC3H13	human ACTB mRNA fragment	n/a	n/a	256.3 ± 27.9 nM	n/a	n/a	Su et al (2022)
WTAP/VIRMA/ZC3H13/HAKAI	human ACTB mRNA fragment	n/a	n/a	214.0 ± 2.6 nM	n/a	n/a	Su et al (2022)
METTL16	UACAGARAA (U6 snRNA, MALAT1)	˜10 µM	>0.4 mM	18 ± 7 µM	126 ± 6 µM	0.07 ± 0.02 min^−1^	Warda et al (2017), Yu et al (2021b), and Breger and Brown (2023)
METTL5/TRMT112	A1832 of 18S rRNA	1.1 ± 0.2 μM (pH 8.0)	1.0 ± 0.2 μM (pH 8.0)	n/a	n/a	13.1 ± 0.8 h^−1^ (pH 8.0)	van Tran et al (2019) and Yu et al (2021b)
ZCCHC4	A4220 of 28S rRNA	n/a	6.7 μM	n/a	n/a	n/a	Ren et al (2019)
PCIF/CAPAM	m^7^GpppAm	3.5 ± 0.71/0.3 ± 0.03 μM (pH 8.0)	0.65 ± 0.05 μM (pH 8.0)	n/a	n/a	0.67 ± 0.01 min^−1^ (pH 8.0)	Akichika et al (2019), Boulias et al (2019), and Yu et al (2021b)
**Readers**							**References**
YTHDC1	G G m^6^A C/G G/A/U	n/a	n/a	0.39 ± 0.071 µM	n/a	n/a	Arguello et al (2019)
YTHDC2	G G m^6^A C/U A/G/C/U	n/a	n/a	321.6 ± 61.9 nM	n/a	n/a	Hsu et al (2017)
YTHDF1	C/G U/G/C m^6^A G/C/U A/G/C/U	n/a	n/a	0.51 ± 0.045/0.13 µM	n/a	n/a	Arguello et al (2019) and Zaccara and Jaffrey (2020)
YTHDF2	A/C/G U/G/C m^6^A G/C/U A/G/C/U	n/a	n/a	0.79 ± 0.018/0.16 µM	n/a	n/a	Arguello et al (2019) and Zaccara and Jaffrey (2020)
YTHDF3	GGm^6^ACU	n/a	n/a	0.14 µM	n/a	n/a	Li et al (2017), Zaccara and Jaffrey (2020), and Zhou et al (2022)
IMP1	UCGGm^6^ACU	n/a	n/a	3.7 ± 0.8 nM	n/a	n/a	Nicastro et al (2023)
IMP1	UCGGACU	n/a	n/a	20.9 ± 4.0 nM	n/a	n/a	Nicastro et al (2023)
**Erasers**			**K_m_ (alpha-ketogluarate)**				**References**
FTO	m^6^A	0.409 ± 0.023 µM	2.88 µM	n/a	n/a	0.296 ± 0.004 (min^−1^)	Jia et al (2008), Jia et al (2011), Ma et al (2012), Mauer et al (2017), Wei et al (2018), and Relier et al (2022)
FTO	m^6^Am	1.34 µM	2.88 µM	n/a	n/a	8.78 (min^−1^)	Mauer et al (2017)
FTO	3meT	0.95 ± 0.12 µM	2.88 µM	n/a	n/a	0.007 ± 0.0002 (min^−1^)	Jia et al (2008) and Ma et al (2012)
FTO	3meU	8.51 ± 3.13 µM	2.88 µM	n/a	n/a	0.115 ± 0.022 (min^−1^)	Jia et al (2008), Jia et al (2011), and Ma et al (2012)
ALKBH5	G(A/G)m^6^ACU	1.38 ± 0.27 µM/192 ± 25 nM	2.5 ± 0.5 µM	n/a	n/a	0.169 ± 0.0106 (min^−1^)	Li et al (2016), Kaur et al (2022), and Peng et al (2023)

The m^6^A modification has also been shown to either promote or limit interactions with proteins directly or indirectly. A set of specific RNA-binding proteins, termed m^6^A readers, can bind specifically to m^6^A-modified RNA and positively or negatively impact the formation of different RNPs at various stages of gene expression regulation (Xu et al, 2014; Hsu et al, 2017; Gilbert & Nachtergaele, 2023; Sikorski et al, 2023).

The m^6^A modification is one of very few RNA modifications that are reversible (Boo & Kim, 2020). Therefore, many modification-associated effects on RNAs and RNPs can be reversed. Two m^6^A demethylases mediate the removal of the modification, also termed m^6^A erasers, fat mass and obesity-associated protein (FTO) or alpha-ketoglutarate–dependent dioxygenase alkB homolog 5 (ALKBH5) (Zhang et al, 2019; Kaur et al, 2022).

The m^6^A RNA modification requires the formation of protein–RNA complexes for its deposition and removal, and it itself modulates the formation of functionally distinct RNPs on modified RNAs by either enabling or preventing the binding of a defined set of protein interactors or other RNAs.

In this review, we aim to give, whenever possible, quantitative descriptions of mechanisms that govern how RNA modifications, specifically m^6^A, are modulated through the dynamic assembly of RNP complexes during the “writing” process and how the modification affects RNA structure, RNP assembly, and function (Table 1). We specifically focus on mechanisms at the basis of RNP assembly and RNA folding related to mRNAs and lncRNAs, not reviewing tRNA and rRNA modifications. The biological roles and regulatory pathways which are modulated by the m^6^A modification and their dysregulation in cancer and other pathologies have been extensively reviewed elsewhere (Barbieri & Kouzarides, 2020; Shi et al, 2020; Huang et al, 2021; Arzumanian et al, 2022; Hong et al, 2022; Boulias & Greer, 2023; Gilbert & Nachtergaele, 2023; Orsolic et al, 2023) and are not part of this review.

### Site-specific deposition of m^6^A modifications on nascent RNA is enabled by protein–RNA and protein–protein interactions

#### The catalytic core enzyme

**Figure 1.**
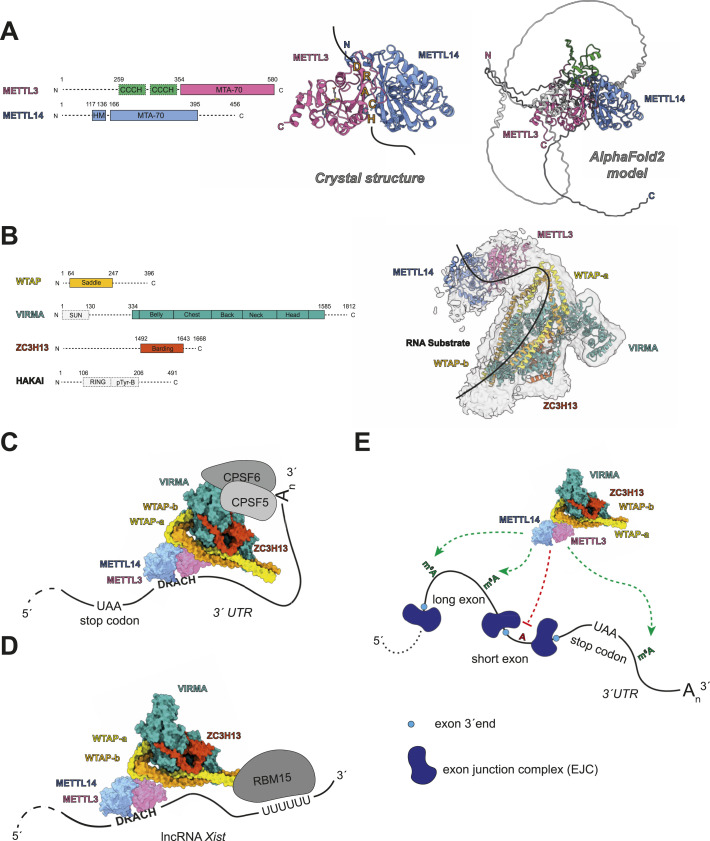
Mechanisms of how the m^6^A core enzyme and its associated regulatory protein domains get recruited to or excluded from different target sites. **(A)** Structure of the m^6^A enzymatic core complex consisting of methyltransferase METTL3 and the critical adapter protein METTL14. Left panel: domain structure of the METTL3 and METTL14 core enzymes. Middle panel: crystal structure of the METTL3/METTL14 complex (MAC) bound to methyl-donor S-Adenosyl methionine (SAM) (gray). The crystal structure contains the METTL3 MTA-70 methyltransferase domain (pink) and the HM and MTA-70 domains of METTL14 (blue); PDB-ID: 5IL1 (Wang et al, 2016). Right panel: AlphaFold 2 model (Mirdita et al, 2022) of the full-length complex between METTL3 and METTL14. Parts of METTL3 and METTL14 that are also present in the X-ray structure are colored in pink and blue, respectively. The CCCH domains of METTL3 are illustrated in green. **(B)** Regulatory subunit m^6^A-METTL-associated complex (MACOM). Left panel: domain structures of adapter proteins WTAP, VIRMA, ZC3H13, and HAKAI. Right panel: Cryo-EM structure of the WTAP, VIRMA, and ZC3H13 complex fitted into a low-threshold cryo-EM density map; PDB-ID: 7VF2 (Su et al, 2022). The METTL3/METTL14 X-ray structure (PDB-ID: 5IL1 (Wang et al, 2016)) is fitted into an additional low-threshold density. A potential binding interface for an RNA substrate (black) is illustrated based on protein-RNA crosslinking data from Su et al (2022). The 5′ to 3′ directionality of the RNA is unknown. **(C)** Model of how the METTL3/METTL14 core enzyme is recruited to mRNA 3′UTRs by VIRMA-meditated interactions with polyadenylation factors CPSF5 and CPSF6; PDB-ID: 5IL1 and 7VF2 (Yue et al, 2018). **(D)** Model of how METTL3/METTL14 is recruited to DRACH motifs on *Xist* lncRNA, mediated by RBM15 binding to U-rich stretches; PDB-ID: 5IL1 and 7VF2 (Patil et al, 2016). **(E)** Model of the exclusion-based mechanism whereby the m^6^A methylation complex cannot bind if the EJC occludes sites; PDB-ID: 5IL1 and 7VF2 (He et al, 2023; Uzonyi et al, 2023). ChimeraX version 1.6 was used for the visualization of experimental and predicted structures (Goddard et al, 2018; Pettersen et al, 2021). Panel (B) was adapted from Su et al (2022) under the Creative Commons CC By license (license: https://creativecommons.org/licenses/by/4.0/).

The correct and successful installation of each m^6^A modification on a specific transcript requires the spatially and temporally coordinated assembly of different dynamic RNPs. At the center of these RNP assemblies is the m^6^A methyltransferase core enzyme METTL3/METTL14, also referred to as m^6^A–METTL complex (MAC) (Liu et al, 2014; Wang et al, 2016; Śledź & Jinek, 2016). METTL3 is the active methyltransferase harboring a binding pocket for the methyl donor S-Adenosyl methionine (SAM), whereas METTL14 is catalytically inactive but crucial for substrate binding and positioning and is required to yield an active enzyme (Fig 1A) (Wang et al, 2016; Śledź & Jinek, 2016; Schöller et al, 2018). Posttranslational methylation of METTL14 R255 has also been demonstrated to positively impact catalytic activity by enhancing RNA binding (Liu et al, 2021).

METTL3 and METTL14 get assembled into a functional enzyme right after translation and posttranslational processing in the cytosol and are subsequently imported into the nucleus as one functional unit, in which only METTL3 carries a nuclear localization signal (Schöller et al, 2018; Han et al, 2022). To ensure a stoichiometric ratio between METTL3 and METTL14 and to maintain m^6^A homeostasis, recent findings show that METTL3 competes with E3 ubiquitin ligase STUB1 for binding to METTL14 and enhances METTL14 stability by preventing its degradation (Zeng et al, 2023).

The functional METTL3/METTL14 enzyme localizes mainly to the nucleus and recognizes a distinct five-nucleotide-long consensus motif, the DRACH motif (D = A,G,U; R = A,G; H = A,C,U), within which the central adenosine base gets methylated (Table 1 and Fig 1A) (Csepany et al, 1990; Liu et al, 2014). In vitro, the core enzyme preferentially methylates single-stranded DRACH motifs (Liu et al, 2014; Meiser et al, 2020). Interestingly, the enzyme displays an increased affinity towards double-stranded RNA in vitro and simultaneously a decreased enzymatic turnover (Qi et al, 2022). The preference for single-stranded motifs hints that the METTL3/METTL14 enzyme has no or only a limited ability to remodel structured RNAs.

Besides modifying RNAs, the METTL3/METTL14 enzyme has also been reported to be active on single-stranded DNA in vitro, which has been linked to DNA repair at UV- or X-ray-induced double-stranded DNA lesions in vivo (Yu et al, 2021a; Qi et al, 2022).

Whereas localization of METTL14 seems to be restricted to the nucleus under physiological conditions, METTL3 is also present in the cytoplasm, where it can promote translation independent of its catalytic activity. METTL3 binds to existing m^6^A marks, directly recruits translation initiation factors CBP80/20 and eIF4E, and facilitates recruitment of eIF3 (Liu et al, 2014; Lin et al, 2016; Schöller et al, 2018).

In summary, the active m^6^A RNA methyltransferase METTL3 forms a functional and catalytic unit with binding partner METTL14 and methylates preferably the short DRACH consensus motif in single-stranded RNA in the nucleus.

#### Co-transcriptional m^6^A deposition

An increasing body of research demonstrates that m^6^A modification by the METTL3/METTL14 enzyme is installed co-transcriptionally (Ke et al, 2017; Knuckles et al, 2017; Slobodin et al, 2017; Louloupi et al, 2018; Lee et al, 2021; Xu et al, 2022). Efficient co-transcriptional RNA modification requires co-localization and/or recruitment of the m^6^A core enzyme to actively transcribing genes on chromatin to facilitate its interaction with the nascent RNA emerging from Pol II.

**Figure 2.**
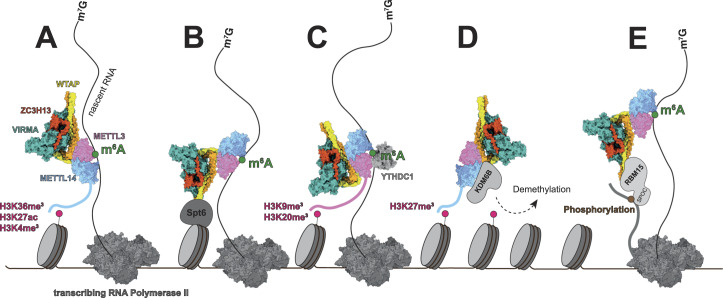
Mechanisms of MAC/ MACOM recruitment to nascent RNA for co-transcriptional RNA modification. The methylation complex is recruited to the nascent RNA by the following interactions/mechanisms. **(A)** Direct interactions between METTL14 and histone marks H3K36me3, H3K27ac, and H3K4me3 (Barbieri et al, 2017; Huang et al, 2019; Dou et al, 2023). **(B)** Direct interactions between WTAP and the transcription elongation factor Spt6 (Akhtar et al, 2021). **(C)** Direct interactions between METTL3 and histone marks H3K9me3 and H3K20me3 and maintained by m^6^A reader YTHDC1 (Xu et al, 2021). **(D)** Direct interactions between METTL14 and histone mark H3K27me3 leads to the recruitment of histone demethylase KDM6B, which directly interacts with METTL14 (Dou et al, 2023). **(E)** Direct interaction between the RBM15 SPOC domain with phosphorylation marks on the C-terminus of RNA polymerase II (Appel et al, 2023). **(A, C, D)** Note that it is not known which parts of the METTL14 (A, D) or METTL3 (C) proteins are interacting with the histone methylation marks. Structures used in this figure are as follows: METTL3/METTL14 (MAC) (PDB-ID: 5IL1), WTAP/ZC3H13/VIRMA (PDB-ID: 7VF2), and human RNA polymerase II (PDB-ID: 3J0K). ChimeraX version 1.6 was used for the visualization of experimental and predicted structures (Goddard et al, 2018; Pettersen et al, 2021).

A potential mechanism to promote spatial proximity between the methylation enzyme and nascent RNAs is through direct or indirect interaction of METTL3 or METTL14 with epigenetic marks on histone tails. METTL14, for instance, directly interacts with the histone H3 trimethylation at Lys 36 (H3K36me3), an epigenetic mark for active transcriptional elongation, and with active histone marks H3K27ac and H3K4me3 (Fig 2A) (Barbieri et al, 2017; Huang et al, 2019; Dou et al, 2023). METTL3, on the other hand, gets recruited to repressive histone marks H3K9m3 and H4K20m3. This recruitment depends on the catalytic activity of METTL3 and is maintained by direct interactions between METTL3 and the nuclear m^6^A reader YTHDC1 (Fig 2C) (Xu et al, 2021).

Furthermore, because of their co-localization to active and repressive histone marks, both METTL3 and METTL14 have been associated with regulating transcriptional activation and repression. METTL14, for example, binds to repressive histone mark H3K27me3 in a DNA- and RNA-independent manner and recruits lysine demethylase 6B (KDM6B) to these sites via a direct interaction. KDM6B can then remove H3K27me3 marks and promote transcriptional activation of the affected genes (Fig 2D) (Dou et al, 2023).

Another study found that co-transcriptional m^6^A modification of chromatin-associated regulatory RNAs (carRNAs) by METTL3 leads to transcriptional repression of the affected genes. Unmodified carRNAs bind and recruit proteins, such as CBP/EP300 and YY1, to promote open chromatin and activate transcription. Once methylated, the m^6^A-marked carRNAs are bound by nuclear m^6^A reader protein YTHDC1 and then targeted for degradation via the nuclear exosome targeting complex, depleting carRNA-associated transcriptional activators from the affected sites (Liu et al, 2020).

A chromatin-independent recruitment of the methyltransferase complex might be facilitated by the C-terminal RGG-rich domain of METTL14, which has the potential to interact with G-quadruplex structures on nascent RNAs. In vitro, enrichment of m^6^A-modified DRACH motifs close to G-quadruplex structures could be demonstrated, but if this mechanism is relevant in the cellular contexts remains to be understood (Yoshida et al, 2022).

#### Target selection by specific recruitment of the core methyltransferase complex to RNA through adapter proteins

Strikingly the number of DRACH motifs in the transcriptome far outnumbers the motifs that are experimentally verified to be methylated in cells. On mRNAs, m^6^A marks are enriched in long exons and introns, close to stop codons, and in the 3′UTRs, whereas DRACH motifs show no specific enrichment (Dominissini et al, 2012, 2013; Meyer et al, 2012; Ke et al, 2017). The mechanism by which the METTL3/METTL14 complex selects or is recruited to these specific sites is still unclear. To date, selectivity in vivo has been proposed to occur via two distinct mechanisms: (1) via specific recruitment of the core methyltransferase complex through adapter proteins or (2) via the exclusion of certain sites through competitive binders.

Within the cell, the METTL3/METTL14 core enzyme interacts with a growing number of protein interactors that recruit the methyltransferase to different cellular locations and prevent or facilitate its interaction with target sites on different RNAs (Bertero et al, 2018; Covelo-Molares et al, 2021).

Precursor-mRNA (pre-mRNA) splicing factor Wilm’s tumor 1-associated protein (WTAP) is frequently found to be associated with the m^6^A core enzyme (Fig 1B) (Liu et al, 2014). WTAP is an important key factor modulating the enzymatic activity of the METTL3/METTL14 complex in vivo by enhancing its RNA-binding ability, but it is dispensable for efficient enzymatic activity in vitro (Ping et al, 2014; Wang et al, 2016; Śledź & Jinek, 2016; Yan et al, 2023). The interaction between the core enzyme and WTAP is at least in part mediated by direct interactions with the N-terminal domain of METTL3 and is not affected by posttranslational phosphorylation on METTL3 (Ping et al, 2014; Schöller et al, 2018; Su et al, 2022). The association of WTAP with the core enzyme is favored in phase-separated states, such as nuclear speckles. This is in line with the observation that the localization of the core enzyme into nuclear speckles is dependent on its association with WTAP (Schöller et al, 2018; Han et al, 2022).

In Drosophila, the WTAP homolog FI(2)d recruits the METTL3/METTL14 complex to actively transcribing genes via direct interactions with the transcription elongation factor and histone chaperone Spt6 (Akhtar et al, 2021). In this context, the core enzyme recruitment to nascent RNAs is facilitated via the adapter protein WTAP, rather than direct interaction with histone marks (Fig 2B). During hepatitis C infection, WTAP relocalizes to the cytosol and recruits the METTL3/METTL14 complex to hepatitis C virus RNA, which promotes viral RNA methylation. A direct interaction between WTAP and the viral RNA seems required for this recruitment, but the details of this interaction remain to be described (Sacco et al, 2022).

Besides recruiting the core enzyme to nuclear speckles and nascent RNAs, WTAP also acts as the main interaction hub for the METTL3/METTL14 enzyme and mediates many crucial interactions with other adapter proteins. One such WTAP-dependent interactor is virilized homolog (VIRMA) (Fig 1B) (Yue et al, 2018; Su et al, 2022). VIRMA itself directly interacts with the polyadenylation factors cleavage and polyadenylation specificity factor subunit 5 (CPSF5) and CPSF6 in an RNA-dependent manner and recruits the METTL3/METTL14/WTAP complex to the 3′ ends of target mRNAs and thereby regulates the preferential installment of the m^6^A modification on 3′UTRs and near stop codons (Fig 1C) (Yue et al, 2018). Another recent publication found that WTAP and VIRMA together also counteract the interaction of the METTL3/METTL14 enzyme with double-stranded DNA and potentially also double-stranded RNA and increase methylation efficiency on single-stranded RNA (Yan et al, 2023).

The composite interaction interface created by WTAP and VIRMA recruits Zinc-finger CCCH-type containing 13 (ZC3H13) protein and E3 ubiquitin ligase HAKAI to the core enzyme (Fig 1B) (Su et al, 2022). ZC3H13 itself is an RNA-binding protein that greatly enhances the RNA-binding affinity of the core enzymes towards RNA in vitro and therefore appears to be an integral partner in regulating m^6^A modification by facilitating the m^6^A RNP assembly (Table 1) (Wen et al, 2018; Su et al, 2022). Furthermore, ZC3H13 knockdown leads to translocation of most of the WTAP, VIRMA, and HAKAI into the cytoplasm, suggesting that its presence is important for maintaining the nuclear localization of these factors, which are critical to regulating the METTL3/METTL14 core enzyme (Wen et al, 2018).

Not much is known about the function of HAKAI in association with the m^6^A methylation machinery, but it has been reported that it has a stabilizing effect on the other components of the m^6^A machinery, including METTL3, METTL14, WTAP, and VIRMA, for which its catalytic activity as E3 ubiquitin ligase is not required (Bawankar et al, 2021).

Because of their frequently observed association and regulatory functions in complex with the METTL3/METTL14 enzyme, the adapter proteins WTAP, VIRMA, HAKAI, and ZC3H13 are referred to as regulatory subunit m^6^A–METTL-associated complex (MACOM) and are considered integral players in regulating m^6^A deposition.

In addition to components of the MACOM, proteomics analysis revealed that WTAP also directly interacts with RNA-binding motif protein 15 (RBM15) and its paralog RBM15B and recruits it to the METTL3/METTL14 core enzyme (Horiuchi et al, 2013). RBM15/15B interacts with U-rich sequences in RNAs, and by this mechanism, recruits the m^6^A core enzyme to specific DRACH motifs on lncRNA *Xist* (Fig 1D) (Patil et al, 2016). Furthermore, the SPOC domain of RBM15 has been demonstrated to bind phosphoserines and to act as a reader of phosphorylation marks on the C-terminal domain of Pol II, which poses another potential mechanism by which the core enzyme gets recruited to nascent RNAs for co-transcriptional modification (Fig 2E) (Appel et al, 2023).

Furthermore, ERK1-mediated phosphorylation of METTL3 S43 has recently been shown to induce m^6^A modification of small nuclear RNA 7SK, by promoting METTL3 release from inhibitor HEXIM (Perez-Pepe et al, 2023). A connected study showed that the 7SK snRNA contains eight m^6^A sites, which are subject to methylation and demethylation by METTL3/METTL14 and ALKBH5, respectively. The presence of methylation at these sites seems to induce a conformational change in 7SK, that favors the release of Pol II transcriptional activator P-TEFb and transcriptional up-regulation (Wang et al, 2023).

#### Target selection via DRACH-motif exclusion through competitive binders

Though a subset of methylated DRACH sites can so far be explained by site-specific recruitment of the core enzyme by adapter proteins to defined sites, it does not give a sufficient explanation for methylation target selection.

Two recent studies suggest an alternative mechanism by which the distinct distribution of m^6^A marks on mRNAs can be partially explained by an exclusion-based mechanism through the binding of the exon junction complex (EJC) on the pre-mRNA (Fig 1E). The EJC thereby masks sites of around 100 nucleotides upstream and downstream of the splice junction and makes them physically inaccessible for the methyltransferase complex. This still permits methylation in longer exons, close to stop codons, and at the 3′UTR and noncoding RNAs, that are not spliced (He et al, 2023; Uzonyi et al, 2023). In addition, both studies find that the METTL3/METTL14 complex does not seem to have any selectivity on which sites to methylate besides the previously mentioned DRACH motif, proposing a mechanism by which mRNA methylation might be guided mainly by excluding or suppressing certain DRACH motifs through physical barriers from being methylated. A similar exclusion-based process has been described in heat shock response. During heat shock, the m^6^A reader protein YTHDF2 localizes to the nucleus and preferentially binds m^6^A marks installed on the 5′UTR of mRNAs and, thereby, prevents their removal by demethylase FTO by masking the methylation marks (Zhou et al, 2015).

As the assembly of the EJC happens after splicing has been completed, those studies also challenge the notion that m^6^A deposition happens immediately on nascent transcripts, as suggested by the direct recruitment of the methylation complex to specific chromatin sites and additional research showing that m^6^A is deposited in nascent RNAs, in introns, and that m^6^A modifications at splice junctions can lead to a more efficient splicing (Louloupi et al, 2018; Xu et al, 2022).

These seemingly contradictory findings may depict a more complex picture of m^6^A target site selection and modification timing by the METTL3/METTL14 enzyme. Certain mechanisms might be limited to certain transcripts, for example, noncoding RNAs, or cellular conditions; in other cases, multiple mechanisms might affect the target selection on the same transcript. Future investigations will consolidate current literature and give an updated model.

In brief, the m^6^A core enzyme METTL3/METTL14 relies on the coordinated association with adapter proteins or the timely assembly of trans-acting machineries for defining site-specific methylation. These interactions are in many cases facilitated by splicing factor WTAP but also occur via direct interactions with other components of the core enzyme.

### Effect of m^6^A modification on RNA structure and protein binding

Through the addition of a methyl group on the base of any given nucleotide, the chemical and physical properties of the affected nucleotides are changed. These changes affect an RNA molecule by either influencing its structure and/or enabling or preventing interactions with cellular interactions partners. Both mechanisms have the potential to facilitate or inhibit the assembly and downstream function of protein–RNA complexes in the cell. In the following sections, we aim to summarize what is known about how altered physiochemical properties of m^6^A-modified RNAs influence their structure and interactions.

#### m^6^A affects RNA structure formation

**Figure 3.**
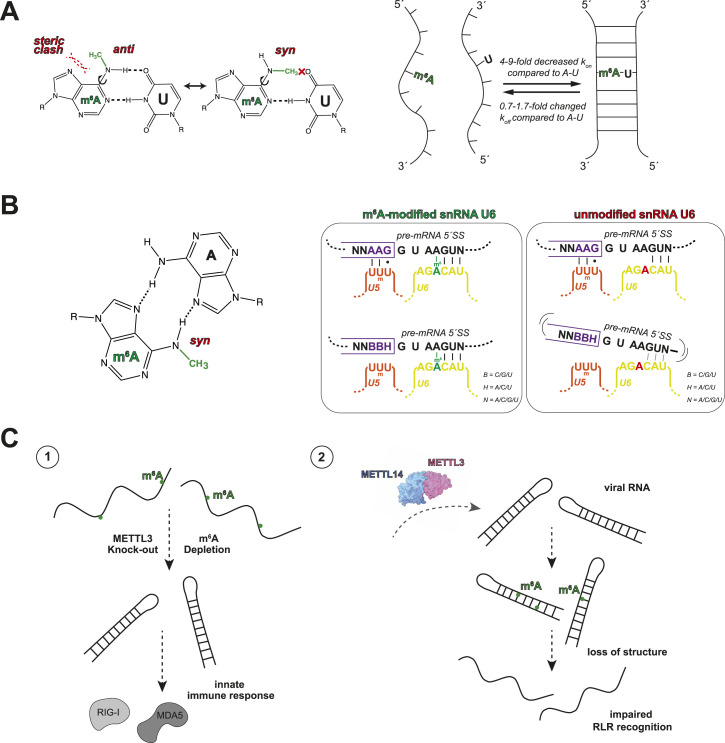
Effects of m^6^A modifications on RNA base-pairing and structure. **(A)** Left panel: stable Watson–Crick base-pairing between m^6^A-U is only possible in the energetically less favorable *anti*-conformation of the m^6^A base. Right panel: two RNA strands containing an m^6^A-U instead of an A-U base-pair have a fourfold to ninefold decreased annealing rate constant but a dissociation rate constant which is not significantly changed by the methylation (Shi et al, 2019). **(B)** Left panel: Hoogsteen–Hoogsteen m^6^A-A base-pair is stabilized compared with Hoogsteen–Hoogsteen A–A (Roost et al, 2015). Right panel: base-pairing between pre-mRNA 5′splice site (5′SS) and modified (green) and unmodified (red) U6 snRNA (yellow) and U5 snRNA (orange). Successful base-pairing between modified U6 snRNA and 5′SS does not require a conserved AAG motif upstream of the 5′SS, but this conserved motif is required with unmodified U6 snRNA for stable base-pairing (Ishigami et al, 2021; Parker et al, 2022). **(C)** Left panel: depletion of m^6^A marks during METTL3 knock-out favors secondary structure formation of otherwise single-stranded endogenous RNAs, which triggers the recognition of these aberrant double-stranded RNAs by RIG-I and MDA5 resulting in an innate immune response (Gao et al, 2020). Right panel: double-stranded virus RNA from vesicular stomatitis virus gets methylated by METTL3, which leads to loss of structure and an impaired RLR recognition and innate immune response (Qiu et al, 2021). Structures used in this figure are as follows: METTL3/METTL14 (PDB-ID: 5IL1). ChimeraX version 1.6 was used for the visualization of experimental and predicted structures (Goddard et al, 2018; Pettersen et al, 2021). Panel (B) was adapted from Ishigami et al (2021) under the Creative Commons CC By license (license: https://creativecommons.org/licenses/by/4.0/).

The m^6^A modifications introduce an additional methyl group at the nitrogen at position six of the nucleobase. The presence of this additional methyl group at the Watson–Crick interface leads to a decrease of the rate constant for duplex association (*k*_*on*_) by ∼fourfold to ninefold for an RNA–RNA or RNA–DNA duplex containing an m^6^A-U/T base pair. In contrast, the rate constant for duplex dissociation (*k*_*off*_) changes only by 0.7–1.7fold (Shi et al, 2019). This means that an RNA duplex structure containing an m^6^A-modified base is less readily and efficiently folded, resulting in a local destabilization of RNA structures. Atomistically, the methyl group on the N^6^ of the modified adenosine base has to adopt the less energetically favorable *anti*-conformation to form a Watson–Crick H^6^--O^4^ hydrogen bond. This results in the destabilization of a double-stranded GGACU by 1 kcal/mol (Fig 3A) (Roost et al, 2015; Shi et al, 2019). This subtle destabilization is sufficient to loosen up RNA duplexes enough to allow the binding of single-stranded RNA-binding proteins or impair cellular checkpoint processes that rely on base complementarity but will most likely only fine-tune interactions rather than act as yes-or-no switches (see below). As a comparison, the Gibbs standard free energies for G-C and A-U Watson–Crick base-pairs are −5.53 and −4.42 kcal/mol, respectively (Vendeix et al, 2009). Therefore, it is also unlikely that the key effects of the m^6^A modification are major m^6^A-induced RNA structural rearrangements.

The destabilizing effect induced by the m^6^A modifications, furthermore, depends on the structural context within the RNA. One study showed that m^6^A can stabilize an m^6^A-U base pair in the presence of Mg^2+^ when it is directly adjacent to a 5′ bulge (Liu et al, 2018). This finding is supported by the fact that m^6^A modifications induce a structural transition in the immediate proximity of the modification. Nucleotides directly 5′adjacent to the modified base tend to adopt a single-stranded conformation, whereas nucleotides directly 3′ to m^6^A are more likely to be base-paired (Roost et al, 2015).

Interestingly, another study showed that the m^6^A modification also has a destabilizing effect on the m^6^A – 8-oxo-G base-pairing at the Hoogsteen interface, leading to a drop in a DNA duplex melting temperature of a 13-base pair-long duplex from 47°C to 44°C. However, by which mechanism this influences nucleic acid folding and which are the potential downstream consequences are still unexplored (Wang et al, 2017).

These subtle changes in the base-paring kinetics can affect biological processes in the cell mainly in two ways (1) by altering processes that are directly dependent on base-pairing or (2) by altering the RNA structure to enable or prevent protein association.

#### Altered base-pairing kinetics directly affect cellular processes that rely on base complementarity

Pre-mRNA splicing in eukaryotes is an essential cellular process that depends, among other things, on base-pairing kinetics. In humans, adenosine 43 (A43) of the U6 snRNA is specifically modified by methyltransferase METTL16 (Pendleton et al, 2017; Warda et al, 2017). A43 is stringently conserved and base-pairs with the 5′-splice site (5′SS) of pre-mRNAs. Interestingly, in higher eukaryotes, which carry the m^6^A modification in U6 snRNA, the 5′SS is less conserved than in lower eukaryotes, especially at the +4 position. Two studies, in *A. thaliana* and *S. pombe*, report that in the presence of the m^6^A in the U6 snRNA, 5′SSs with an A in position 4 are favored as the modification slightly stabilizes the m^6^A-A base pair by 0.7 kcal/mol as compared with an A–A base pair. A 5′SS with a U at position 4, on the other hand, gets destabilized by 0.5–1.7 kcal/mol and preferentially base-pairs with the loop 1 region of U5 snRNA instead of the U6 snRNA (Roost et al, 2015; Ishigami et al, 2021; Parker et al, 2022) (Fig 3B). These small thermodynamic changes in base-pairing stability induced by the m^6^A modification lead to a relaxation in the 5′ exon constraints and allow for increased protein diversity in higher eukaryotes.

Followed by successful splicing, base-pairing kinetics is also crucial for mRNA decoding during translation. The presence of an m^6^A-modified nucleotide influences efficient base-pairing between the codon on the mRNA and the anticodon on the cognate tRNA leading to only near-cognate codon-anticodon interactions, thereby slowing down proper tRNA accommodation. Under high-accuracy conditions (1.3 mM MgCl_2_), this results in a reduction of the Michaelis–Menten parameter (k_cat_/K_m_)_pep_ for peptide bond formation at m^6^AAA codons by a factor of 18 as compared with AAA codons (Choi et al, 2016). This effect of the m^6^A modification during decoding depends on the position and sequence context within the codon. Another study recently confirmed these findings by showing that m^6^A does not prevent canonical codon–anticodon base-pairing but favors alternative conformations leading to a lower stability and enhancing the tRNA drop-off. This consequently results in a smaller number of ribosomes that complete the decoding process, resulting in a less efficient translation of the affected transcripts (Jain et al, 2023).

A related study connected the reduced translational efficiency caused by the m^6^A modification to the transcriptional speed of Pol II (Slobodin et al, 2017). The researchers showed that faster transcription rates result in fewer methylation marks on mRNAs, resulting in a more efficient translation of the affected transcripts and vice versa. This mechanism allows an indirect coupling between transcription and translation in an m^6^A-dependent manner in eukaryotes.

The propensity of m^6^A-modified nucleotides to alter base-pairing kinetics also reduces the formation of endogenous double-stranded RNAs. Upon the deletion of the m^6^A methyltransferase METTL3 in murine fetal liver, RNAs, which are normally highly m^6^A-modified and show a low folding propensity, form double-stranded structures. These aberrantly formed endogenous double-stranded RNAs lead to an anomalous activation of the innate immune response by activating pattern recognition receptors such as RIG-I and MDA5 (Fig 3C) (Gao et al, 2020).

In addition, the RNA duplex destabilizing effect of m^6^A can also be hijacked by viruses. Infection by double-stranded virus RNA vesicular stomatitis virus leads to translocation of m^6^A methyltransferase METTL3 to the cytoplasm and an increase in m^6^A marks on viral RNAs. This reduces the proportion of double-stranded viral RNA and thereby the efficiency by which it is sensed and cleared by retinoic acid-inducible gene I-like receptors (RLRs) (Fig 3C) (Qiu et al, 2021).

Overall, the m^6^A modification leads to subtle changes in the RNA folding kinetics that result in small changes in the local RNA structure. Those changes carefully fine-tune cellular processes sensitive to base-pairing such as splicing and translation, and can affect the immunogenicity of endogenous or pathogenetic RNAs.

#### m^6^A-mediated RNA structural changes create new protein binding sites

The altered base-pairing characteristics induced by the m^6^A modification are also associated with RNA structural rearrangements that facilitate protein–RNA interactions.

This is exemplified by the interaction of splicing factors heterogeneous nuclear proteins C and G (hnRNP-C and hnRNP-G) with pre-mRNAs and lncRNAs (Liu et al, 2015, 2017).

**Figure 4.**
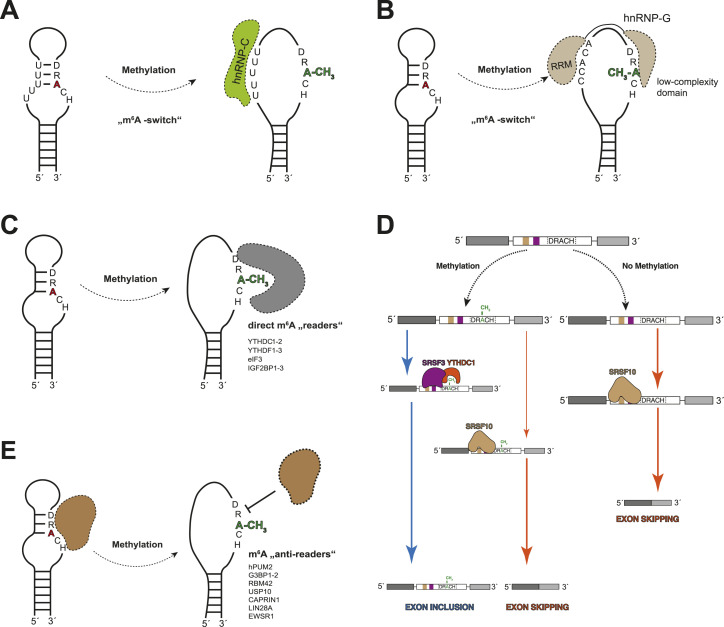
Direct and indirect effects of m^6^A modifications on reader protein binding. Panels (A, B) show indirect m^6^A readers, whereas panels (C, D, E) show direct m^6^A readers or “anti-readers.” **(A)** “m^6^A-switch” mechanism 1: methylation of DRACH motifs by the m^6^A writer complex within RNA hairpins leads to the release of U-rich stretches, which can then be bound by RNA-binding protein hnRNP-C (Liu et al, 2015). **(B)** “m^6^A-switch” mechanism 2: Methylation of DRACH motifs by the m^6^A writer complex within RNA hairpins leads to the release of the DRACH motif from the RNA structure, which allows RNA-binding protein hnRNP-G to bind the single-stranded AGRAC motifs (R = A/G) with its low-complexity domain (Liu et al, 2017). The RRM domain of hnRNP-G can bind CC(A/C)-rich sequences independent of the m^6^A modification (Heinrich et al, 2009). **(C)** m^6^A modifications by the m^6^A writer complex create a new binding epitope for m^6^A reader proteins such as YTHDC1-2 and YTHDF1-3. **(D)** m^6^A marks within exons in pre-mRNA can be bound by m^6^A reader YTHDC-1 (orange), which recruits splicing factor SRSF3 (purple) that promotes exon inclusion and prevents binding of splicing factor SRSF10 (brown), which would promote exon skipping in the absence of the m^6^A mark. **(E)** m^6^A modifications by the m^6^A writer complex can also disrupt protein binding interfaces. The affected class of proteins is termed m^6^A “anti-readers.”

The protein hnRNP-C binds U-rich single-stranded RNA stretches in pre-mRNA and lncRNAs. These can be masked within double-stranded RNA structures. Upon modification, the A-U Watson-Crick base-pair gets destabilized, thereby releasing U-rich binding sites for hnRNP-C. The ability of hnRNP-C to bind such U-rich stretches in pre-mRNA in an m^6^A-dependent manner subsequently leads to the generation of hnRNP-C-dependent splicing variations (Fig 4A) (Liu et al, 2015).

The protein hnRNP-G is a regulator of alternative splicing and preferentially binds single-stranded AGRAC motifs (R = A/G), via its intrinsically disordered or low-complexity domain. Strikingly, the hnRNP-G binding motif strongly overlaps with the m^6^A DRACH motif. Methylation of the DRACH motif can release the hnRNP-G binding motifs from double-stranded RNA structures allowing hnRNP-G to bind (Liu et al, 2017). The binding of hnRNP-G to its motif happens independently of the m^6^A modification in single-stranded RNAs. Similarly to the binding by hnRNP-C, the m^6^A-dependent accessibility of the consensus motif enables hnRNP-G–specific regulation of alternative splicing (Fig 4B) (Zhou et al, 2019). The RNA-recognition motif (RRM) domain of hnRNP-G, on the other hand, binds single-stranded CC(A/C)-rich motifs m^6^A-independently (Heinrich et al, 2009), thereby providing additional binding specificity.

Heterogeneous nuclear protein A2/B1 (hnRNPA2B1) binds AGG and UAG motifs via its two RNA-recognition motif domains through a similar mechanism as hnRNP-G. hnRNPA2B1 binds these motifs in a subset of pri-miRNAs and interacts with Dgcr8, a component of the microprocessor complex, thereby promoting miRNA processing (Alarcón et al, 2015a; Wu et al, 2018).

The release of specific single-stranded recognition sequences by m^6^A-induced base-pairing changes has been termed an “m^6^A switch.”

An example of how altered base-pairing kinetics can prevent the formation of a protein–RNA complex are box C/D small nucleolar RNAs (snoRNAs). Box C/D snoRNAs are the core component of the Box C/D snoRNP, which installs 2′-O-methylation marks in rRNA during ribosome biogenesis. For correct assembly of this multicomponent RNP, the Box C and D consensus motifs on the snoRNA have to fold into a characteristic kink-turn conformation to allow the binding of the protein core components. The Box D motif contains a GAC motif which is potent for m^6^A methylation by the METTL3/METTL14 complex. An m^6^A modification within the Box D motif interferes with formation of a structurally critical trans Hoogsteen-sugar A-G base-pair, disrupting the kink-turn fold. The kink-turn fold is required for binding of the Box C/D-associated proteins. Therefore, the Box C/D snoRNP cannot be assembled in the presence of an m^6^A modification in the Box D motif (Huang et al, 2017).

In summary, the subtly altered base-pairing kinetics resulting from the presence of an m^6^A modification also affects the availability of single-stranded RNA-binding interfaces. Therefore, the modification can fine-tune the binding of specific RNA-binding proteins and affect cellular processes such as splicing or miRNA processing.

#### The m^6^A modification creates new binding epitopes for proteins

Besides altering base-pairing properties, which directly affect the RNA structure and trans-interactions with other RNAs and proteins, the additional methyl group also introduces a new binding epitope for protein interaction partners. The m^6^A modifications can influence the protein interactome of a given modified RNA either by (1) serving as an “m^6^A-switch” as described in the previous section, (2) by providing m^6^A-specific binding pockets (Dominissini et al, 2012, 2013) or (3) by weakening the interaction with a protein binding partner (Arguello et al, 2017).

##### YTH domain-containing proteins

YTH domain-containing proteins are the best-known family of m^6^A-interacting proteins, or m^6^A readers. This protein family has been associated with almost all cellular processes linked to the m^6^A modifications to date, including transcriptional regulation, pre-mRNA splicing, RNA nuclear export, translation regulation, and RNA stability and decay (Boulias & Greer, 2023).

Proteins of this family bind the m^6^A modification specifically through their conserved YTH-domain (Fig 4C). Within the YTH domain, two crucial aromatic residues form the m^6^A binding pocket. Removal of either residue leads to loss of m^6^A binding (Theler et al, 2014; Xu et al, 2014; Zhu et al, 2014). The YTH domain is characteristically flanked by two intrinsically disordered regions at the N- and C-terminus (Sikorski et al, 2023).

YTHDC1 is the only exclusively nuclear YTH-domain-containing m^6^A reader protein. In contrast to the other YTH domain-containing m^6^A readers, YTHDC1 shows a sequence preference towards the canonical DRACH motif, with a preference for C after the m^6^A residue and purines at the n-1 and n-2 positions around the methylation site (Table 1). Because of its nuclear localization, YTHDC1 is involved in almost all nuclear processes that involve m^6^A-modified RNAs, including chromatin remodeling, transcriptional regulation, X-chromosome inactivation, mRNA processing, and nuclear mRNA export (Widagdo et al, 2022).

YTHDC1 can be recruited to co-transcriptionally m^6^A-modified nascent RNAs and recruits the histone H3K9me3 demethylase KDM3B by an unknown mechanism. This leads to the demethylation of H3K9 and, thereby, the removal of a repressive histone mark (Li et al, 2020).

Furthermore, YTHDC1 has the propensity to initiate phase separation through its arginine-rich C-terminal disordered domain (Cheng et al, 2021; Lee et al, 2021). One study showed that m^6^A modifications deposited on enhancer RNAs are bound by YTHDC1 and form m^6^A–enhancer RNA/YTHDC1 condensates. These condensates can undergo mixing with BRD4 coactivator condensates and facilitate the formation of transcriptional activator condensates (Lee et al, 2021). Together with splicing factor hnRNPG, YTHDC1 can also prevent premature transcription termination by binding to co-transcriptionally installed m^6^A-marks on 5′-ends of RNAs thereby preventing binding of the integrator complex (Xu et al, 2022). During pre-mRNA splicing, YTHDC1 is furthermore involved in alternative splicing regulation. YTHDC1 is recruited to m^6^A-modified exons and recruits splicing factor SRSF3 via direct interactions between its C-terminus and the C-terminus of SRSF3, resulting in exon inclusion. At the same time, binding of YTHDC1 inhibits the binding of splicing factor SRSF10 and thereby interferes with SRSF10-associated exon skipping. In the absence of an m^6^A modification within a given exon, SRSF10 can bind and promote exon skipping (Fig 4D) (Xiao et al, 2016). Finally, at the 3′-end of mRNAs, YTHDC1 can interfere with alternative polyadenylation leading to longer 3′UTRs (Chen et al, 2022).

YTHDC1 stays associated with mature m^6^A-modified mRNA to facilitate their nuclear export through the direct interaction with splicing factor and nuclear export adapter SRSF3. SRSF3, on the other hand, can interact with the nuclear export receptor NXF1, which facilitates the nuclear export of m^6^A-modified mRNA through protein–protein interactions (Roundtree et al, 2017).

The m^6^A marks on lncRNA Xist are also recognized and bound by YTHDC1. This interaction with Xist is crucial for X-chromosomal inactivation and gene silencing (Patil et al, 2016). Mechanistically, YTHDC1 binds to m^6^A-modified highly conserved AUCG tetraloops in the A-repeats, which leads to partial melting of the hairpin and modulation of the RNA structure (Jones et al, 2022).

The YTHDF paralogs YTHDF 1–3 are preferentially localized to the cytosol. Two contradictory models have been proposed regarding the function of these three different proteins. The canonical model assigns each YTHDF paralog-defined functions and targets, whereas a more recent model proposes that all three proteins have redundant functions and targets in mRNA degradation (Zaccara & Jaffrey, 2020).

Within the canonical model, YTHDF1 enhances the translation of m^6^A-modified transcripts by binding m^6^A marks at the stop codon and 3′ UTRs of mRNAs. YTHDF1 then directly interacts with and recruits translation initiation factor eIF3 to promote cap-dependent translation (Wang et al, 2015).

YTHDF2, on the other hand, is involved in mediating mRNA decay (Wang et al, 2014). To promote m^6^A-modified mRNA decay, YTHDF2 binds the m^6^A marks on mRNAs and either directly recruits the CCR4–NOT deadenylation complex (Du et al, 2016) or interacts with HRSP12, which in turn recruits the RNaseP/MRP endoribonuclease (Park et al, 2019).

In the nucleus, YTHDF2 is also involved in the clearance of R-loop structures. R-loops are three-stranded nucleic acid structures, consisting of a DNA:RNA hybrid and a single-stranded DNA, that are formed at the transcription bubble during transcription. RNA in R-loop structures can be m^6^A-modified by the METTL3/METTL14 methyltransferase, which leads to the recruitment of reader YTHDF2 to promote mRNA degradation, and R-loop clearance (Abakir et al, 2020; Kang et al, 2021).

Lastly, YTHDF3 is a modulator of YTHDF1 and YTHDF2 functions. YTHDF3 enhances translation through direct interaction with YTHDF1 (Shi et al, 2017). Similarly, YTHDF3 also interacts with YTHDF2 and seems to be involved in mRNA decay through this interaction (Shi et al, 2017). In addition, YTHDF3 binds and recognizes stress-induced newly methylated mRNA and drives their translocation into stress granules during oxidative stress (Anders et al, 2018). It is important to point out that a more recent study claims that m^6^A marks on mRNAs have only a limited effect on their translocation into stress granules (Khong et al, 2022).

The more recent model proposes that all three paralogs share similar targets and mediate mRNA degradation via association with CNOT, which is a scaffolding subunit of the CCR4vNOT deadenylase complex (Zaccara & Jaffrey, 2020).

The detailed functions and an attempt to consolidate the two existing models have recently been reviewed (Sikorski et al, 2023).

In contrast to the other members of the YTH domain family, YTHDC2 comprises several well-folded domains, including the YTH domain. YTHDC2 can interact with the small ribosomal subunit close to the mRNA entry and exit sites via its YTH and R3H domains. Therefore, YTHDC2 is thought to facilitate efficient translation by bridging m^6^A-marked mRNAs with the ribosome (Kretschmer et al, 2018). Furthermore, it can recruit 5′-3′ exonuclease XRN1 via direct interaction through its ankyrin domain and potentially promote mRNA decay (Kretschmer et al, 2018).

In summary, YTH domain-containing proteins bind m^6^A-modified RNA via similar mechanisms but lead to different downstream effects. How substrate specificity is achieved outside of the modification itself still needs to be better understood and will need further investigation to elucidate distinct functions and functional redundancies.

##### Non-YTH domain m^6^A readers

Non-YTH domain-containing m^6^A readers recognize and bind m^6^A-modified RNAs via alternative modes of recognition and are often dependent on the cellular context.

One such m^6^A reader is the translation initiation factor eIF3. eIF3 can directly read m^6^A marks in the 5′ UTRs of mRNA and recruits the 43S pre-initiation complex to the translation start site. Through this mechanism, eIF3 can initiate m^6^A-dependent/cap-independent translation initiation under stress conditions (Meyer et al, 2015).

Insulin-like growth factor 2 mRNA-binding proteins 1, 2, and 3 (IGF2BP1/2/3) are a family of distinct m^6^A readers, which specifically bind and recognize m^6^A modifications within the GG(m^6^A)CU motif (Huang et al, 2018). IGF2BPs bind the m^6^A modifications via their K homology (KH) domain, though how exactly the binding specificity is achieved is still unclear. In contrast to YTHDF proteins, IGF2BP proteins promote the stability of their mRNA targets in the cytosol and promote their translation.

A novel pair of m^6^A reader proteins is FMR1 and its paralogs FXR1 and FXR2. These proteins have been known to bind RNAs with the consensus motifs ACUG/U or U/A/GGGA, which show strong overlap with the METTL3/METTL14 DRACH consensus sequence (Ascano et al, 2012). Indeed, FXR1 was recently identified as a sequence context-specific m^6^A reader (Edupuganti et al, 2017). Furthermore, FXR1 has been described to bind to m^6^A marks in nascent RNAs and recruit DNA 5-methylcytosine dioxygenase TET1 to active chromatin loci leading to DNA demethylation and reprogrammed chromatin accessibility (Deng et al, 2022).

IMP1, a recently described m^6^A reader, interacts with the modification via a dedicated hydrophobic platform, enabling a high-affinity interaction (Table 1). This interaction is sequence-independent but is embedded into the methylation-independent sequence preference of IMP1, GGAC, which has significant similarity with the METTL3/METTL14 DRACH motif (Nicastro et al, 2023). The methyl group within the binding sequence results only in a small change in the on-rate from *k*_*on*_ = 1.3 × 10^5^ ± 0.1 × 10^5^ M^−1^s^−1^ to *k*_*on*_ = 8.7 × 10^4^ ± 0.1 × 10^4^ M^−1^s^−1^ but a more significant decrease in the off-rate from *k*_*off*_ = 2.7 × 10^−3^ ± 0.4 × 10^−3^ s^−1^ to *k*_*off*_ = 3.2 × 10^−4^ ± 0.7 × 10^−4^ s^−1^, highlighting how the modification can specifically increase the lifetime of a protein–RNA complex.

Lastly, proline-rich coiled-coil 2A (PRRC2A) is a recently discovered m^6^A reader critical for male spermatogenesis. It recognizes m^6^A marks within DRACH sequences, but little is known yet on the molecular details that govern this interaction (Wu et al, 2019; Tan et al, 2023).

#### Interactions negatively affected by the m^6^A modification

In the previous sections, we described examples in which the modification facilitates interactions, but RNP assembly can similarly be negatively affected by the m^6^A modification. Protein–RNA interactions that are negatively affected by the m^6^A modification include human Pumilio 2 (hPUM2), stress granule proteins G3BP1, G3BP2, RBM42, USP10, CAPRIN1, and RNA-binding proteins LIN28A, and EWSR1 (Fig 4E). Such proteins are termed m^6^A “anti-readers.”

One of the best-described m^6^A “anti-readers” is single-stranded RNA-binding protein hPUM2 (Vaidyanathan et al, 2017). hPUM2 consists of eight PUF repeats, each recognizing and making specific interactions with one base within its consensus motif. The presence of an m^6^A-modified base within this sequence weakens the binding of hPUM2. This effect is amplified with increasing m^6^A modifications, showing a quantitative negative effect of hPUM2 binding to its target in an m^6^A-dependent manner. The presence of m^6^A-modified bases within the 11-nucleotide-long hPUM2 recognition sequence (CCUGUAUAUAU) results in an increase of the K_D_ from 0.14 to 0.34 nM with one m^6^A and 5.3 nM with three m^6^A bases (Vaidyanathan et al, 2017).

A proteome-wide screen has identified stress granule proteins G3BP1, G3BP2, RBM42, USP10, and CAPRIN1 as m^6^A “anti-readers” (Arguello et al, 2017). The abundance of m^6^A “anti-readers” in the stress granule proteome suggests that m^6^A-marked RNAs are not specifically targeted into stress granules or might even be actively excluded (Edupuganti et al, 2017; Khong et al, 2022). The exact mechanism through which these stress granule proteins sense the m^6^A modification remains to be elucidated.

LIN28A is a potent cellular inhibitor of pri-miRNA processing and antagonizes m^6^A reader hnRNPA2B1, which is associated with promoting pre-miRNA processing (Viswanathan et al, 2008; Alarcón et al, 2015a). LIN28A has been termed an m^6^A “anti-reader” as it shows decreased enrichment on m^6^A-marked RNAs, hinting a reduced affinity to modified RNAs (Sun et al, 2019). In the current model, LIN28A binds unmodified pri-miRNAs and antagonizes the installation of the m^6^A mark by METTL3/METTL14 to prevent binding of hnRNPA2B1 and the maturation of pri-miRNAs.

In summary, m^6^A “anti-readers” are a group of proteins whose interactions with their RNA targets are negatively affected by the presence of the m^6^A modification. Only a few examples of m^6^A “anti-readers” are known, and only for a small fraction of those the mechanism of modification recognition is described.

### Reversing m^6^A-facilitated RNA interactions by “erasing” the modification

Another feature of m^6^A RNA modifications is that they can be removed during the life cycle of an RNA. The methyl group removal is achieved through one of two characterized m^6^A demethylases: FTO or ALKBH5 (Zhang et al, 2019; Kaur et al, 2022).

Both FTO and ALKBH5 are alpha-ketoglutarate-dependent dioxygenases and require oxygen, alpha-ketoglutarate, and Fe(II) to demethylate RNA. FTO localizes mainly in the nucleus and nuclear speckles but is also present in the cytosol (Jia et al, 2011; Wei et al, 2018). ALKBH5, on the other hand, localizes mainly into nuclear speckles (Thalhammer et al, 2011; Zheng et al, 2013). ALKBH5 acts only on m^6^A-modified bases, whereas FTO also acts on N^6^,2′-O-dimethyladenosine (m^6^Am), N^1^-methyladenosine (m^1^A), 3-meThymine (3-meT), and 3-meUracil (3meU)-modified nucleotides (Table 1) (Jia et al, 2011; Mauer et al, 2017; Wei et al, 2018; Relier et al, 2022).

The main substrates for FTO are internal m^6^A and m^6^Am modifications on mRNA and snRNA and m^1^A modifications on tRNA (Wei et al, 2018). The influence of specific sequences or RNA structural motifs on demethylation remains largely unclear (Han et al, 2010; Zhang et al, 2019). A large screening for cellular interaction partners showed that FTO interacts with a wide variety of proteins involved in transcription, RNA binding, splicing, DNA repair, and chromatin remodeling, some of which might also be involved in determining substrate specificity (Covelo-Molares et al, 2021). The splicing factor SFPQ, for example, has been identified as a binding partner of FTO and associates with the demethylase via its C-terminal domain. Available data show that SFPQ binds the RNA motif CUGUG and recruits FTO to m^6^A sites nearby to promote demethylation (Song et al, 2020). SFPQ-directed demethylation accounts for only 20% of FTO-mediated demethylation targets.

ALKBH5 seems to act only on internal m^6^A-modified nucleotides. Furthermore, it shows a sequence preference toward m^6^ACU motifs, which notably overlaps with the known METTL3/METTL14 DRACH motif (Kaur et al, 2022). ALKBH5 interacts with factors involved in chromatin remodeling, suggesting that similar to m^6^A methyltransferase METTL3/METTL14, it also localizes to actively transcribing genes. A recent article showed that ALKBH5 associates with the newly identified m^6^A reader RBM33, which activates the demethylase by suppressing SUMOylation and regulates the demethylation target selection (Yu et al, 2023). ALKBH5 further associates with components of the TREX complex, the EJC, and components involved in pre-mRNA splicing and miRNA-mediated RNA decay (Covelo-Molares et al, 2021).

So far, only little is known about FTO and ALKBH5 in their function as m^6^A demethylases. Recent years have unveiled the structural and catalytic basis for the demethylation, and important binding partners have been identified. Nevertheless, target specificity, interaction modes, specific functions, and potential functional redundancies are poorly understood. It is also unclear if specific RNA molecules can get methylated and demethylated multiple times. Future research in these directions will shed more light on the importance of reversing RNA modifications in the coming years.

## Conclusions and Outlook

Protein–RNA assemblies play an integral part in many cellular processes, and the coordinated installment of RNA modifications can modulate their assembly. In this review, we aimed to summarize how m^6^A modifications, from their deposition until their removal, are functionally coupled to RNA folding and protein–RNA complex formation.

The process of the modification itself is a highly coordinated chain of events that relies on protein–RNA and protein–protein complex assembly. It starts with the site-specific and direct recruitment of the m^6^A core enzyme METTL3/METTL14 to sites of active transcription. This process is mainly mediated by direct protein–protein interactions between the core enzyme and defined histone marks or the C-terminus of Pol-II. The close spatial proximity between the enzymatic machinery and the nascent RNA allows for co-transcriptional installation of the modification (Huang et al, 2019; Wu et al, 2020; Xu et al, 2021). The core enzyme methylates preferentially single-stranded DRACH motifs within the newly synthesized RNA (Wang et al, 2016; Śledź & Jinek, 2016; Qi et al, 2022). Interestingly, only a subset of DRACH motifs gets modified in vivo, with a bias towards long exons and introns, and stop codons and 3′UTRs in mRNA (Dominissini et al, 2012, 2013). Two complementary mechanisms can partially explain this discrepancy: (1) site-specific recruitment of the core enzyme by protein–protein interactions with adapter proteins and (2) masking of DRACH motifs by competitive trans-acting factors (Ping et al, 2014; Yue et al, 2018; Su et al, 2022; He et al, 2023; Uzonyi et al, 2023). For site-specific recruitment, the METTL3/METTL14 core enzyme is targeted to specific DRACH motifs by different adapter proteins, including the MACOM complex components: WTAP, VIRMA, ZC3H13, and HAKAI. Complementary to protein-based recruitment, two very recent studies proposed a mechanism that excludes certain DRACH motifs through competitive binders, such as the exon–junction complex, and also challenge the notion that the modification is mainly installed co-transcriptionally (He et al, 2023; Uzonyi et al, 2023).

After installation, m^6^A changes the base-pairing kinetics, by weakening Watson–Crick m^6^A-U base-pairs and stabilizing Hoogsteen–Hoogsteen/Watson–Crick m^6^A-A base pairs (Roost et al, 2015; Shi et al, 2019). This alters the local RNA-folding kinetics, which modulates the binding of proteins such as hnRNP-G and hnRNP-C to newly available binding sites on the modified RNA (Liu et al, 2015, 2017; Zhou et al, 2019). Furthermore, the additional methyl group also introduces a new or modified binding epitope for RNA-binding proteins. YTH domain-containing proteins are the most common specific m^6^A readers and bind RNA preferentially in the presence of the modification.

Writers and readers can also cooperate. For example, both the m^6^A reader YTHDC1 and the METTL3/METTL14 core enzyme localize to chromatin, and can thereby cooperate to maintain a repressive chromatin state at murine retroviral elements (Xu et al, 2021).

Different writers can also compete with each other. For example, during pri-miRNA processing, the m^6^A modifications allow for regulated competition between inhibitors and facilitators, as is the case for m^6^A “anti-reader” LIN28A and m^6^A reader hnRNPA2B1 (Alarcón et al, 2015a; Sun et al, 2019).

Through the examples illustrated in this review, it becomes clear that RNA modifications, here specifically m^6^A, are regulated by the coordinated assembly of protein–RNA assemblies and play an integral role in modulating the assembly of diverse sets of protein–RNA complexes and thereby affect their downstream functions. Although more and more roles are being proposed for m^6^A in different pathways and the interactome is constantly expanding, only a few and often very specific cases describe the mechanistic details of m^6^A-mediated regulation in protein–RNA assemblies.

We still do not have high-resolution structural information on the RNA bound to MAC or MAC/MACOM. Furthermore, questions such as m^6^A target-site selection, how the modification and the process of modification affect RNA folding, and temporal information on the coordinated action of m^6^A writers, readers, and erasers remain to be answered. Also, little is still known about the mechanistic details of how m^6^A modification is functionally coupled to cellular processes such as transcription and the effect of RNA modification on co-transcriptional splicing (Martinez & Gilbert, 2018). Quantitative methods such as single-molecule fluorescence microscopy and other emerging biophysical and structural approaches in vitro and in vivo (Gor & Duss, 2023) will be key to understanding and tracking these multiple-step processes and to uncovering and understanding the crosstalk between RNA modification, RNA folding, and protein–RNA complex formation and function.
